# Glutamine-Linked Cellular Stress Responses in Viral Infection: Mechanisms, Crosstalk, and Future Perspectives

**DOI:** 10.3390/ijms27114717

**Published:** 2026-05-23

**Authors:** Ngan Thi Kim Pham, Quang Duy Trinh, Hiroshi Ushijima, Shihoko Komine-Aizawa, Kazuaki Yoshimune

**Affiliations:** 1 Department of Applied Molecular Chemistry, College of Industrial Technology, Nihon University, Chiba 274-0072, Japan; pham.thikimngan@nihon-u.ac.jp (N.T.K.P.); yoshimune.kazuaki@nihon-u.ac.jp (K.Y.); 2 Division of Microbiology, Department of Pathology and Microbiology, Nihon University School of Medicine, Tokyo 173-8610, Japan; ushijima-hiroshi@jcom.home.ne.jp (H.U.); aizawa.shihoko@nihon-u.ac.jp (S.K.-A.)

**Keywords:** glutamine metabolism, integrated stress response (ISR), virus–host interactions, cellular stress response, ER stress, autophagy, oxidative stress, metabolic stress, viral replication, viral entry

## Abstract

Glutamine is the most abundant amino acid in human plasma and tissues and plays essential roles in cellular metabolism, biosynthesis, and redox homeostasis. Beyond these canonical functions, glutamine availability and utilization have emerged as key regulators of multiple cellular stress responses, including the integrated stress response, endoplasmic reticulum stress, metabolic checkpoint signaling, and autophagy. During viral infection, host glutamine metabolism is frequently reprogrammed to meet the energetic and biosynthetic demands of viral replication, thereby inducing or reshaping glutamine-linked stress pathways. Increasing evidence indicates that these stress responses are not merely secondary consequences of infection but actively influence key stages of the viral life cycle, including viral entry, genome replication, protein synthesis, and host antiviral responses. In this review, we summarize current advances in understanding how glutamine metabolism regulates cellular stress responses in the context of both viral and non-viral infections, and how these pathways, in turn, modulate viral pathogenesis and host defense. We discuss the context-dependent roles of glutamine-linked stress signaling in either promoting viral replication or restricting infection, depending on viral species, host cell type, and metabolic conditions. Finally, we highlight emerging concepts and unresolved questions, including the potential of targeting glutamine metabolism and associated stress pathways as host-directed antiviral strategies. A deeper understanding of the interplay between glutamine metabolism, cellular stress responses, and viral infection may provide new insights into disease mechanisms and inform the development of novel therapeutic approaches.

## 1. Introduction

Glutamine is the most abundant free amino acid in human plasma and tissues and plays central roles in cellular metabolism, biosynthesis, and redox homeostasis [1,2,3]. Traditionally classified as a non-essential amino acid, glutamine becomes conditionally essential under conditions of physiological stress, rapid cell proliferation, or nutrient limitation, reflecting its critical importance in maintaining cellular homeostasis [1,3]. As a versatile metabolic substrate, glutamine contributes carbon and nitrogen for nucleotide, amino acid, and lipid biosynthesis, while also supporting mitochondrial metabolism through anaplerotic replenishment of tricarboxylic acid (TCA) cycle intermediates [2,4]. In addition, glutamine-derived glutamate is a key precursor for glutathione (GSH) synthesis, thereby contributing to the maintenance of intracellular redox balance [5].

Beyond these canonical metabolic functions, accumulating evidence indicates that glutamine availability is tightly linked to the regulation of cellular stress responses. Fluctuations in glutamine levels can activate multiple stress signaling pathways, including the integrated stress response (ISR), endoplasmic reticulum (ER) stress and unfolded protein response (UPR), metabolic checkpoint signaling mediated by AMP-activated protein kinase (AMPK) and mechanistic target of rapamycin (mTOR), as well as autophagy [6,7,8,9]. These pathways collectively enable cells to adapt to nutrient deprivation, oxidative stress, and biosynthetic imbalance through coordinated regulation of gene expression, protein synthesis, and cellular metabolism [7,10]. Importantly, these pathways are extensively coordinated and collectively influence cellular fate under conditions of metabolic imbalance.

Viral infections impose substantial metabolic demands on host cells, requiring efficient production of viral genomes, proteins, and membrane components. To support these processes, many viruses actively reprogram host cellular metabolism, including glutamine uptake and utilization, thereby reshaping intracellular metabolic fluxes [9,11,12]. Such metabolic reprogramming often resembles nutrient stress conditions and can lead to activation of glutamine-linked stress pathways. These stress responses, in turn, may either support viral propagation by enhancing biosynthetic capacity or limit infection through activation of host defensive programs [11,13,14,15]. For example, activation of the ISR can modulate host translational control, ER stress can influence protein folding capacity required for viral assembly, and autophagy can either degrade viral components or provide membrane platforms for replication complexes [6,16].

In addition to virus-driven metabolic reprogramming, emerging evidence suggests that pre-existing metabolic stress conditions, such as glutamine insufficiency, hypoxia, or nutrient limitation, can influence cellular susceptibility to viral infection. Under these conditions, stress-induced alterations in host cell physiology, including changes in receptor expression, membrane dynamics, and protein quality control systems, may create a cellular environment that differentially influences viral entry and replication depending on the balance between adaptive stress signaling and antiviral responses [8,15,17,18,19]. These observations highlight a bidirectional relationship between glutamine metabolism and viral infection, in which metabolic state and stress signaling not only respond to infection but also actively influence the course of infection.

Importantly, these pathways are not merely passive consequences of infection but actively participate in host–virus interactions by either limiting viral propagation or being exploited to support distinct stages of the viral life cycle [9,13,16,20].

In this review, we provide a comprehensive overview of the mechanisms by which glutamine metabolism regulates cellular stress responses and how these pathways are engaged during viral and non-viral infections [9,12]. We further discuss the dual roles of glutamine-linked stress signaling in promoting or restricting viral replication, depending on the cellular and metabolic context. Finally, we highlight emerging concepts and unresolved questions, including the potential of targeting glutamine metabolism and associated stress pathways as host-directed therapeutic strategies.

## 2. Glutamine Biology as a Regulator of Cellular Stress

### 2.1. Glutamine Metabolism and Homeostasis

Glutamine homeostasis is tightly regulated through coordinated control of cellular uptake, intracellular synthesis, and metabolic utilization [3,21]. Circulating glutamine is transported into cells primarily via sodium-dependent neutral amino acid transporters, including SLC1A5 (also known as ASCT2) and members of the SLC38 family [21,22]. These transport systems are dynamically regulated according to cellular metabolic demands and are frequently upregulated in rapidly proliferating or metabolically active cells.

Intracellularly, glutamine can be synthesized de novo from glutamate and ammonia by glutamine synthetase (GS), whereas its catabolism is initiated by glutaminase (GLS), which converts glutamine to glutamate [23]. Glutamate is subsequently metabolized to α-ketoglutarate via glutamate dehydrogenase (GLUD1) or aminotransferases, thereby feeding into the TCA cycle [24]. This process, commonly referred to as glutaminolysis, serves as a major anaplerotic pathway that replenishes TCA cycle intermediates and sustains mitochondrial bioenergetics.

Beyond its role in energy production, glutamine contributes to multiple biosynthetic pathways. It provides nitrogen for the synthesis of nucleotides and non-essential amino acids and supports hexosamine biosynthesis, which contributes to protein glycosylation and signal transduction processes [25]. In addition, glutamine-derived glutamate is a critical precursor for GSH, the major intracellular antioxidant, thereby linking glutamine metabolism to redox homeostasis [5].

Glutamine metabolism is highly sensitive to cellular and environmental conditions, including nutrient availability, energy demand, oxidative stress, hypoxia, and infection-associated metabolic reprogramming. Under physiological and stress-associated conditions such as tissue growth, differentiation, hypoxia, or nutrient limitation, glutamine supply may become insufficient, leading to metabolic stress. For example, cells residing in hypoxic or nutrient-restricted microenvironments, including early developmental tissues, frequently experience fluctuations in glutamine availability that require metabolic reprogramming and stress adaptation [26]. Thus, glutamine homeostasis is not static but dynamically regulated to match cellular demands and environmental constraints.

### 2.2. Glutamine Sensing and Activation of Stress Pathways

At the molecular level, glutamine exhibits substantial conformational flexibility and hydrogen-bonding capacity, characteristics that may influence its interactions with metabolic enzymes, transporters, and signaling proteins. Ab initio analyses of glutamine side-chain conformations have demonstrated the energetic adaptability of its molecular configurations, supporting its functional versatility in diverse cellular environments [27]. These physicochemical features may contribute to the sensitivity of glutamine-dependent metabolic and stress-sensing pathways to fluctuations in nutrient availability.

To maintain cellular homeostasis, cells possess sophisticated mechanisms to sense changes in glutamine availability and translate these fluctuations into downstream signaling responses. One of the primary sensors of amino acid insufficiency is general control nonderepressible 2 (GCN2), a kinase activated by the accumulation of uncharged transfer RNAs under conditions of amino acid deprivation [8,28]. Activation of GCN2 leads to phosphorylation of eukaryotic initiation factor 2α (eIF2α), resulting in global attenuation of protein translation while selectively promoting the translation of stress-responsive genes, including activating transcription factor 4 (ATF4). This signaling axis constitutes a central component of the ISR, enabling cells to conserve resources and restore metabolic balance.

In parallel, glutamine availability also influences ER homeostasis, and nutrient imbalance can engage UPR signaling pathways that coordinate proteostasis and cellular adaptation to nutrient stress [7]. Insufficient glutamine can impair protein folding capacity by altering redox conditions and limiting biosynthetic precursors required for proper protein maturation. These perturbations activate the UPR, particularly through the protein kinase RNA-like ER kinase (PERK) pathway, which converges on eIF2α phosphorylation [7]. This convergence highlights an important point of integration between nutrient sensing and proteostasis.

Glutamine levels additionally regulate metabolic checkpoint signaling mediated by mechanistic target of rapamycin complex 1 (mTORC1) and AMP-activated protein kinase (AMPK) [10,29,30]. Adequate glutamine availability promotes mTORC1 activation, supporting anabolic processes such as protein and lipid synthesis. Conversely, glutamine deprivation suppresses mTORC1 activity and activates AMPK through energy stress signaling, thereby shifting cellular metabolism toward catabolic and energy-conserving pathways [10,29].

Together, these sensing mechanisms enable cells to rapidly detect and respond to fluctuations in glutamine availability by coordinating metabolic adaptation with stress signaling. Importantly, these pathways do not function independently but instead form a coordinated regulatory system linking nutrient availability, energy status, and protein homeostasis.

### 2.3. Redox and Biosynthetic Stress Induced by Glutamine Imbalance

Disruption of glutamine homeostasis can lead to profound metabolic and redox disturbances. Because glutamine is a major precursor for GSH synthesis, its depletion reduces intracellular GSH levels and compromises the cell’s antioxidant capacity, resulting in the accumulation of reactive oxygen species (ROS) [5,31]. Elevated ROS levels can damage proteins, lipids, and nucleic acids, thereby exacerbating cellular stress and activating stress-responsive signaling pathways.

In addition to redox imbalance, glutamine deficiency impairs biosynthetic processes. Reduced availability of glutamine-derived nitrogen limits nucleotide synthesis, affecting DNA replication and RNA transcription. Similarly, decreased production of TCA cycle intermediates compromises lipid biosynthesis and other anabolic pathways, leading to a state of biosynthetic insufficiency [2,25]. Together, these alterations generate conditions associated with energy limitation, oxidative stress, and impaired macromolecule synthesis.

Importantly, these stress conditions are not merely detrimental but also serve as signals that activate adaptive pathways, including the ISR, UPR, and autophagy [7,8,32]. Through these mechanisms, cells attempt to re-establish intracellular balance by reprogramming metabolism, reducing biosynthetic demand, and enhancing recycling of intracellular components. However, prolonged or severe glutamine deficiency may overwhelm these adaptive responses, resulting in cellular dysfunction or death.

**Figure 1:**
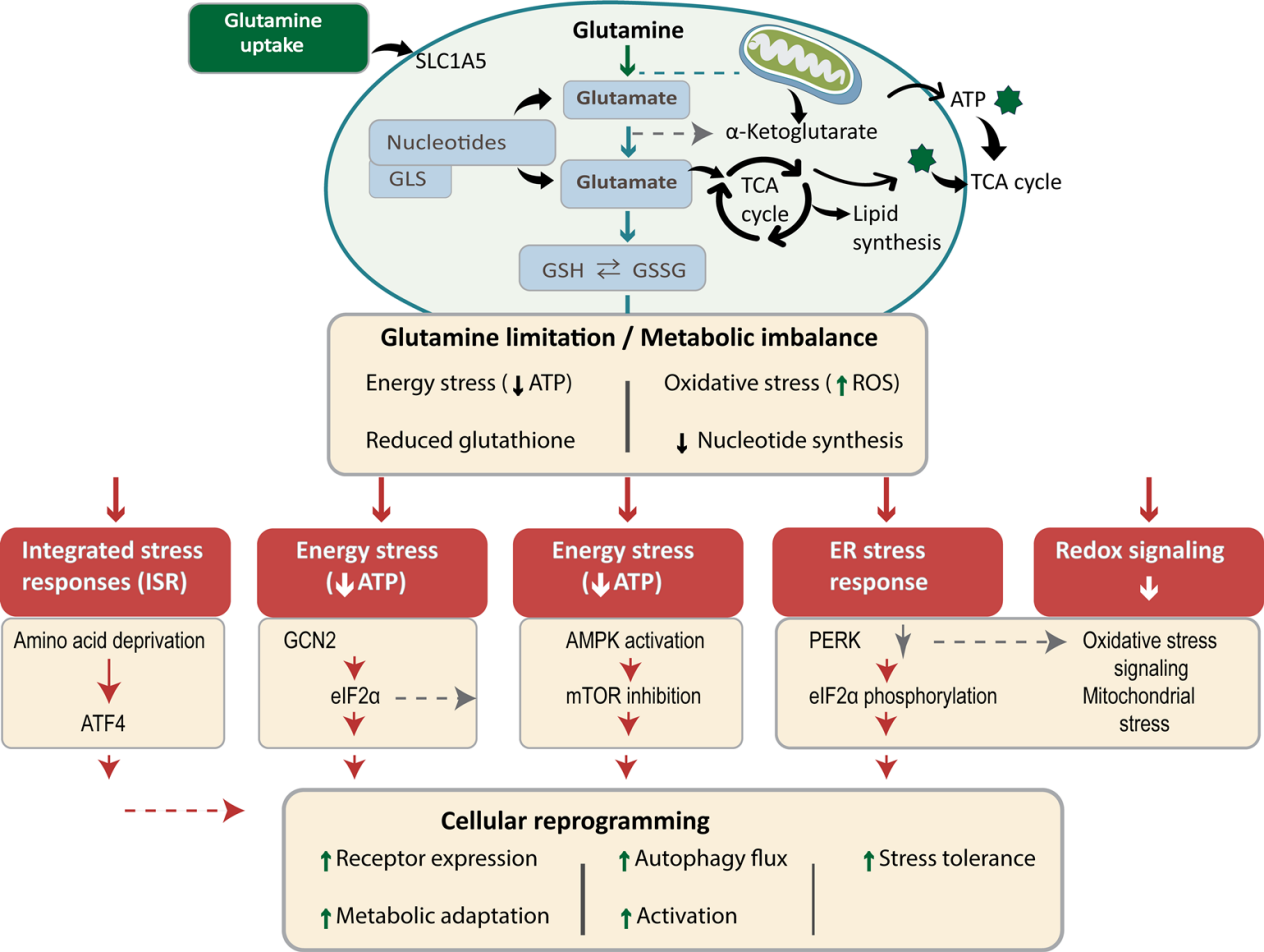
Glutamine metabolism and glutamine-linked cellular stress responses. Glutamine supports mitochondrial metabolism, biosynthesis, and redox homeostasis through its conversion into glutamate and tricarboxylic acid (TCA) cycle intermediates. Under conditions of glutamine insufficiency or metabolic imbalance, disruption of these processes induces oxidative and biosynthetic stress, leading to activation of interconnected stress pathways, including the integrated stress response (ISR), endoplasmic reticulum (ER) stress/unfolded protein response (UPR), AMP-activated protein kinase (AMPK)-mechanistic target of rapamycin (mTOR) signaling, and autophagy. These pathways collectively coordinate metabolic adaptation and cellular homeostasis. Arrows indicate metabolic flow, activation, or signaling progression. Dashed arrows indicate indirect or crosstalk-mediated effects, whereas green upward arrows indicate increased or adaptive cellular responses. Created with Adobe Illustrator, v.25.

A schematic overview of glutamine metabolism and glutamine-linked cellular stress responses is presented in Figure 1.

The metabolic and redox perturbations induced by glutamine imbalance serve as upstream signals that engage multiple stress-responsive pathways. In the following section, we examine in detail how these pathways are activated and coordinated under conditions of glutamine limitation.

## 3. Glutamine-Linked Cellular Stress Pathways

### 3.1. Integrated Stress Response

The ISR is a central adaptive signaling pathway that enables cells to respond to nutrient deprivation and other stress conditions [8,28,33,34,35,36]. Glutamine insufficiency represents a potent trigger of the ISR, primarily through activation of the kinase GCN2, which senses amino acid deficiency via accumulation of uncharged transfer RNAs [8,37]. Activated GCN2 phosphorylates eIF2α, resulting in global attenuation of cap-dependent protein translation while selectively promoting the translation of stress-responsive mRNAs, including ATF4 [8,28,38]. To overcome ISR-mediated translational suppression, several viruses have evolved antagonistic mechanisms that interfere with eIF2α signaling or promote eIF2α dephosphorylation. For example, certain viral proteins recruit host phosphatase complexes, including protein phosphatase 1 (PP1), to reverse eIF2α phosphorylation and sustain viral protein synthesis during infection [39,40].

ATF4 functions as a master regulator of stress-responsive gene expression, inducing pathways involved in amino acid transport, redox balance, metabolic reprogramming, and stress-associated autophagy [28,32,38,41,42]. Under conditions of glutamine limitation, ATF4-dependent transcription promotes restoration of amino acid homeostasis while simultaneously reducing biosynthetic demand [42,43]. In addition, ISR signaling is closely linked to autophagy induction, providing an alternative source of intracellular nutrients through lysosomal degradation of cellular components [32,38].

Importantly, ISR signaling does not operate in isolation but is closely coordinated with other cellular stress programs, including ER stress and metabolic checkpoint signaling. However, persistent or excessive ISR activity may promote apoptotic signaling through downstream effectors such as CHOP, highlighting the dual role of ISR in cell survival, cell death, and translational regulation during viral infection [8,14,28].

### 3.2. Endoplasmic Reticulum Stress and the Unfolded Protein Response

The ER plays a critical role in protein folding and maturation, processes that are highly sensitive to cellular metabolic status and are tightly coordinated by the UPR [7]. Glutamine deprivation can disrupt ER homeostasis by limiting the availability of biosynthetic precursors and altering intracellular redox balance, thereby promoting the accumulation of misfolded proteins [7].

This condition activates the UPR, a multi-branch signaling system mediated by three principal ER stress sensors: PERK, IRE1, and ATF6 [7,44,45]. Among these, PERK is particularly relevant in the context of glutamine-linked stress, as it converges on eIF2α phosphorylation, thereby intersecting with ISR signaling. This convergence functionally links nutrient sensing to proteostasis control.

Activation of the UPR induces PERK-mediated eIF2α phosphorylation, IRE1-dependent signaling, and ATF6 processing, thereby promoting transcriptional and translational programs that enhance protein folding capacity and proteostasis. ER stress–associated upregulation of the molecular chaperone GRP78/BiP has also been implicated in viral infection processes. Cell surface–localized GRP78 can function as a co-receptor or auxiliary host factor for SARS-CoV-2 entry through interactions with the viral spike protein and ACE2, highlighting the mechanistic interplay between ER stress signaling and viral infection [46,47]. In addition, ER stress can stimulate autophagy as a mechanism to alleviate proteotoxic stress [7,48]. However, prolonged or unresolved ER stress may trigger apoptotic pathways, contributing to cellular dysfunction.

The interplay between glutamine metabolism and ER stress underscores the importance of metabolic status in maintaining proteostasis, particularly under conditions of nutrient limitation.

### 3.3. Metabolic Stress Signaling: AMPK and mTOR

Cellular energy status is tightly regulated by metabolic checkpoint pathways, primarily mediated by AMP-activated protein kinase (AMPK) and the mechanistic target of rapamycin (mTOR). Glutamine deprivation can impair mitochondrial metabolism and reduce ATP production, leading to AMPK activation [10,29]. Activated AMPK functions as a cellular energy sensor that restores energy homeostasis by promoting catabolic pathways that generate ATP while inhibiting anabolic pathways that consume ATP.

One of the key downstream effects of AMPK activation is inhibition of mTOR complex 1 (mTORC1), a central regulator of cell growth and biosynthesis. mTORC1 activity is highly sensitive to amino acid availability, including glutamine, which contributes to its activation through multiple mechanisms, including regulation of intracellular amino acid pools and signaling through Rag GTPases [29,30,36]. Under glutamine-deficient conditions, suppression of mTORC1 leads to decreased protein synthesis and enhanced autophagy.

The AMPK–mTOR axis therefore serves as a central regulatory interface between glutamine metabolism and cellular adaptation, coordinating energy homeostasis, biosynthetic regulation, and stress responses.

### 3.4. Autophagy as an Adaptive Response

Autophagy is a conserved cellular process that degrades and recycles intracellular components to maintain metabolic homeostasis [6,49,50]. It is strongly induced under conditions of nutrient deprivation, including glutamine insufficiency [6]. Activation of autophagy is mediated by multiple upstream signals, including AMPK activation and mTORC1 inhibition, as well as contributions from ISR and ER stress pathways [45,48,49,50].

Through the formation of double-membrane autophagosomes, cellular macromolecules and organelles are delivered to lysosomes for degradation, releasing amino acids, lipids, and other metabolites that can be reused for energy production and biosynthesis [6]. In the context of glutamine limitation, autophagy serves as a key nutrient-recycling mechanism that replenishes intracellular nutrient pools.

Beyond its metabolic role, autophagy also participates in cellular quality control by removing damaged organelles, including mitochondria, thereby limiting oxidative stress. However, the biological consequences of autophagy depend on context, as excessive or dysregulated autophagy may contribute to cell death.

### 3.5. Integration of Stress Networks

Although individual stress pathways are often described separately, they function as a highly coordinated signaling system that shapes cellular responses to metabolic perturbations. Glutamine deprivation simultaneously influences multiple signaling axes, including ISR, ER stress, AMPK–mTOR signaling, redox regulation, and autophagy, creating a coordinated adaptive response.

These pathways are extensively linked at molecular and functional levels. For example, phosphorylation of eIF2α represents a convergence point between ISR and ER stress signaling, while AMPK activation promotes autophagy and suppresses mTOR-driven anabolic processes. In parallel, oxidative stress can exacerbate ER stress and further activate stress-responsive transcriptional programs [7,8,10].

This networked architecture dynamically adjusts cellular responses to fluctuating metabolic conditions. However, it also creates opportunities for dysregulation, particularly under prolonged or severe stress, where adaptive responses may transition to maladaptive outcomes such as apoptosis or senescence.

Understanding the integration of these pathways is essential for interpreting how glutamine-linked stress responses influence cellular physiology and pathological processes, including viral infection.

The glutamine-linked stress pathways described above are frequently engaged during viral infection, either as a consequence of virus-induced metabolic reprogramming or as pre-existing cellular states that influence infection outcomes. In the following section, we examine how viruses exploit and modulate these stress networks to support viral propagation and persistence.

## 4. Viral Exploitation of Glutamine-Linked Stress Networks

Viruses are obligate intracellular parasites that depend extensively on host cellular metabolism to support the synthesis of viral genomes, proteins, and membrane structures. Because they lack autonomous metabolic machinery, viruses actively reprogram host metabolic pathways to generate a favorable intracellular environment for replication. Among host nutrients, glutamine has emerged as a critical substrate that supports viral infection through its roles in bioenergetics, biosynthesis, and redox homeostasis [11,12].

Viral infection is frequently accompanied by extensive metabolic remodeling that resembles cellular nutrient stress. The high biosynthetic demand required for viral genome replication, protein synthesis, and membrane biogenesis imposes substantial pressure on host metabolic networks. In response, infected cells often increase glutamine uptake and glutaminolysis through upregulation of glutamine transporters and metabolic enzymes, thereby enhancing α-ketoglutarate production to sustain TCA cycle activity and anabolic metabolism [51,52,53]. These metabolic changes not only support viral replication but also perturb cellular homeostasis, leading to activation of glutamine-linked stress pathways, including ISR, ER stress signaling, metabolic checkpoint regulation, and autophagy.

Importantly, these stress responses are not merely passive consequences of infection but represent dynamic regulatory networks that can either restrict viral replication or be co-opted by viruses to facilitate different stages of their life cycle [13,14,16]. In addition, the pre-existing metabolic state of the host cell, including conditions of glutamine insufficiency, hypoxia, or nutrient limitation, may further influence cellular susceptibility to infection and the outcome of virus–host interactions [12,13].

In this section, we first examine the dependence of viral replication on host glutamine metabolism (Section 4.1), followed by a detailed discussion of how viruses interact with specific glutamine-linked stress pathways (Section 4.2, Section 4.3, Section 4.4 and Section 4.5).

### 4.1. Viral Dependence on Host Glutamine Metabolism

Many viruses exhibit a pronounced dependence on host glutamine metabolism to sustain efficient replication. Glutamine contributes to viral propagation through multiple interconnected mechanisms, including fueling mitochondrial metabolism, supporting nucleotide biosynthesis, and maintaining intracellular redox balance [12,20,51].

A central role of glutamine during viral infection is its function as an anaplerotic substrate. Through glutaminolysis, glutamine is converted to glutamate and subsequently to α-ketoglutarate, replenishing TCA cycle intermediates that are continuously consumed during biosynthetic reactions. This replenishment supports ATP production and provides metabolic precursors required for lipid synthesis and nucleotide generation, both of which are essential for the formation of viral replication complexes and genome synthesis [20,52]. In rapidly replicating RNA viruses, which require efficient and timely production of viral components, this glutamine-dependent metabolic support is particularly critical.

Consistent with this, multiple viruses actively enhance glutamine uptake and utilization. For example, infection with Human cytomegalovirus (HCMV) has been shown to increase glutamine consumption to sustain TCA cycle activity and biosynthetic flux, and inhibition of glutamine metabolism markedly impairs viral replication [52]. Similarly, dengue virus and influenza A virus induce metabolic reprogramming that includes increased glutamine utilization, thereby supporting anabolic pathways required for replication [51,54]. In these contexts, upregulation of glutamine transporters and glutaminase activity further reinforces glutaminolysis and metabolic adaptation. For example, adenoviral E4ORF1 protein promotes MYC-dependent metabolic reprogramming, thereby enhancing glutamine utilization and anabolic pathways to facilitate efficient viral replication [53].

Beyond its role in bioenergetics and biosynthesis, glutamine metabolism also contributes to maintaining cellular redox homeostasis through the generation of GSH. Viral infection is often associated with increased oxidative stress due to mitochondrial dysfunction and heightened metabolic activity. Adequate glutamine availability supports GSH synthesis, thereby buffering ROS and allowing infected cells to tolerate the metabolic burden imposed by viral replication [5,31]. Conversely, disruption of glutamine metabolism can exacerbate oxidative stress, which may either impair viral replication or trigger stress responses that viruses can exploit.

Collectively, these observations indicate that glutamine metabolism is frequently co-opted by viruses to meet the energetic and biosynthetic demands of infection while maintaining cellular conditions compatible with viral replication. At the same time, perturbations in glutamine availability contribute to the activation of cellular stress pathways described in Section 3, thereby linking metabolic reprogramming to stress signaling. The following sections examine how viruses interact more directly with these glutamine-associated stress pathways and how such interactions influence viral life cycles.

**Figure 2:**
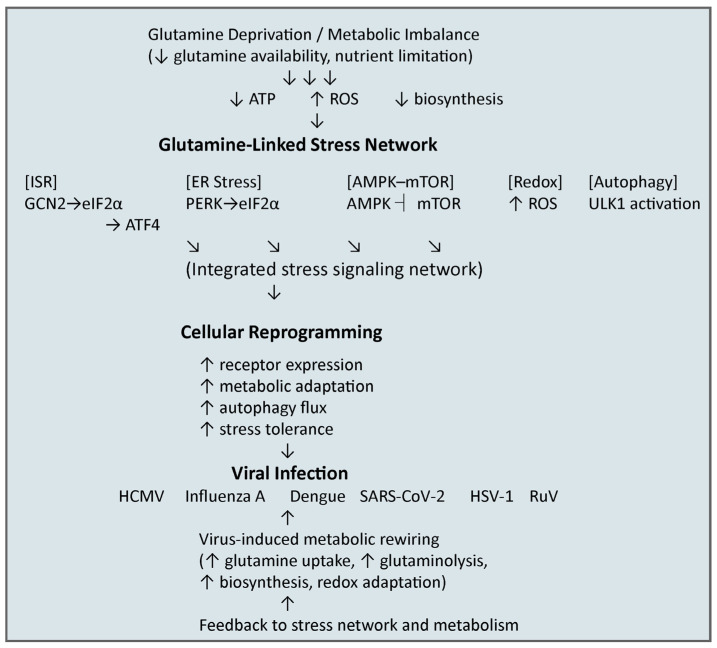
Glutamine deprivation–induced cellular stress networks and their roles in viral infection. Glutamine deprivation or metabolic imbalance activates multiple interconnected stress pathways, including the integrated stress response (ISR), ER stress, redox stress, AMP-activated protein kinase (AMPK)–mechanistic target of rapamycin (mTOR) signaling, and autophagy. Activation of these pathways alters cellular metabolism, protein synthesis, and stress adaptation, thereby influencing viral replication and host susceptibility to infection. Viruses can exploit or modulate these glutamine-linked stress networks to support different stages of their life cycle, establishing a bidirectional relationship between metabolic stress and viral infection. Representative viruses associated with these processes include human cytomegalovirus (HCMV), influenza A virus, dengue virus, severe acute respiratory syndrome coronavirus 2 (SARS-CoV-2), herpes simplex virus 1 (HSV-1), and rubella virus (RuV). Arrows indicate activation or signaling progression, whereas the symbol ⊣ indicates inhibitory regulation (e.g., AMPK inhibits mTOR signaling). Created with Adobe Illustrator, v.25.

A schematic overview of glutamine deprivation–induced cellular stress networks and their roles in viral infection is presented in Figure 2.

### 4.2. Integrated Stress Response in Viral Infection

The ISR is a central signaling pathway that links nutrient availability to translational control and cellular adaptation. During viral infection, ISR activation is frequently observed as a consequence of metabolic stress, accumulation of viral RNA, and perturbations in protein homeostasis [8,14,28,34,55]. Glutamine deprivation represents an important upstream trigger of ISR activation, primarily through activation of GCN2, which senses amino acid insufficiency and promotes phosphorylation of eIF2α [8,28].

Phosphorylation of eIF2α leads to global attenuation of cap-dependent protein translation, thereby limiting cellular energy expenditure and reducing biosynthetic demand. At the same time, selective translation of stress-responsive transcripts, including ATF4, is enhanced, promoting adaptive gene expression programs that regulate amino acid metabolism, redox balance, and cellular survival [28,43]. In the context of viral infection, this translational reprogramming has complex and context-dependent consequences.

On one hand, ISR activation can restrict viral replication by suppressing host translational machinery required for viral protein synthesis, while also modulating antiviral signaling pathways [14,55,56]. Many viruses rely heavily on cap-dependent translation, and inhibition of this process can impair viral propagation. In addition, ISR-associated transcriptional programs may enhance cellular stress tolerance and antiviral defenses [14,56]. On the other hand, numerous viruses have evolved strategies to evade, modulate, or exploit ISR signaling, including maintaining translation of viral mRNAs or counteracting eIF2α-mediated translational suppression [14,55]. For example, HSV-1 ICP34.5 protein recruits PP1 to promote eIF2α dephosphorylation, thereby restoring translation and facilitating viral protein synthesis during infection [39,40]. Similarly, several RNA viruses, including Seneca Valley virus, can suppress host antiviral signaling and stress-associated innate immune responses through interactions between viral non-structural proteins and host regulatory pathways, thereby facilitating viral replication and immune evasion [57,58].

Glutamine-linked metabolic stress further modulates ISR dynamics during infection. Reduced glutamine availability can amplify ISR activation, thereby influencing the outcome of virus–host interactions. In certain contexts, enhanced ISR signaling may favor viral replication by promoting cellular adaptation and survival, enabling sustained viral production. Conversely, excessive or prolonged ISR activation may lead to apoptotic signaling, limiting viral spread.

Notably, ISR signaling is closely interconnected with other stress pathways, including ER stress and autophagy, which are also engaged during viral infection. This integration enables dynamic coordination of cellular adaptation pathways during infection. The outcome of ISR activation therefore depends on multiple factors, including viral species, host cell type, and the metabolic state of the infected cell.

Emerging evidence suggests that metabolic conditions characterized by glutamine insufficiency may influence viral susceptibility through ISR-mediated mechanisms. For instance, stress-induced modulation of translational control, cellular proteostasis, and membrane dynamics may alter the efficiency of viral entry, replication, or protein synthesis in a cell type-dependent manner, although the underlying mechanisms remain incompletely understood. In addition, nutrient availability and metabolic stress conditions can significantly influence viral susceptibility and replication efficiency by altering cellular metabolism, stress signaling, and host antiviral responses [59,60].

Collectively, these findings highlight ISR as a key interface between glutamine metabolism and viral infection. By integrating nutrient sensing with translational control and stress-adaptive signaling, ISR dynamically influences the balance between antiviral defense and viral exploitation in a context-dependent manner, thereby serving as a critical determinant of infection outcomes.

### 4.3. Endoplasmic Reticulum Stress in Viral Infection

ER stress is a common feature of viral infection, reflecting the substantial burden placed on host protein folding and processing machinery during viral protein synthesis [7,14,44,61]. Many viruses rely on the ER for synthesis, folding, and post-translational modification of structural and non-structural proteins, leading to perturbations in ER homeostasis and activation of the UPR [7,14,44].

The UPR is mediated by three principal signaling branches, PERK, IRE1, and ATF6, which collectively function to restore ER homeostasis by attenuating protein synthesis, enhancing protein folding capacity, and promoting degradation of misfolded proteins. Among these, the PERK pathway is particularly relevant in the context of glutamine-linked stress, as it converges with ISR signaling through phosphorylation of eIF2α, thereby integrating nutrient sensing with proteostasis control [7,44]. This convergence enables coordinated regulation of translational attenuation and adaptive gene expression during viral infection.

Glutamine metabolism plays an important role in maintaining ER function. Adequate glutamine availability supports biosynthetic processes required for protein synthesis and contributes to redox balance through GSH production. Under conditions of glutamine deprivation, reduced biosynthetic capacity and impaired redox homeostasis can disrupt protein folding, leading to accumulation of unfolded or misfolded proteins and activation of ER stress signaling. Increased ROS further exacerbate ER stress, reinforcing the link between glutamine metabolism and proteostasis. Emerging evidence further suggests that excessive lipid ROS accumulation and ferroptosis-associated pathways may contribute to redox-dependent cellular injury during viral infection, although their relationships with glutamine-linked stress responses remain incompletely understood [62,63].

During viral infection, ER stress exerts both antiviral and proviral effects. Activation of the UPR can restrict viral replication by limiting protein synthesis and promoting degradation of viral proteins. However, many viruses have evolved mechanisms to selectively modulate UPR signaling to enhance protein folding capacity, lipid biosynthesis, and membrane remodeling required for replication [44,64,65,66,67,68,69]. For example, flaviviral non-structural proteins NS4A and NS4B remodel ER membranes and interact with host stress signaling pathways to facilitate replication complex formation and persistent infection [70]. In addition, modulation of PERK–eIF2α signaling may allow viruses to balance host translational suppression with continued synthesis of viral proteins.

ER stress is also closely interconnected with other glutamine-linked stress pathways. It can promote autophagy as a mechanism to alleviate proteotoxic stress, while also interacting with metabolic checkpoint signaling and redox regulation. These interactions contribute to the formation of an integrated stress network that is dynamically regulated during viral infection [64,71].

Importantly, metabolic conditions associated with nutrient insufficiency may influence ER stress responses prior to or during infection. In such contexts, pre-activation of stress pathways may alter cellular susceptibility to viral entry and replication by affecting protein folding capacity, membrane composition, and receptor processing. For example, metabolic stress induced by glucose limitation has been shown to enhance rubella virus infection in human first-trimester trophoblast-derived cells, highlighting the sensitivity of these cells to nutrient-dependent stress conditions [19]. Although this observation was linked to glucose restriction, glucose and glutamine metabolism are closely interconnected through coordinated regulation of energy production, biosynthesis, and redox homeostasis. Therefore, alterations in glucose availability may indirectly influence glutamine-dependent stress responses and cellular susceptibility to viral infection.

Together, these observations highlight ER stress as a key interface between glutamine metabolism and viral infection. Through its integration with ISR, redox signaling, and autophagy, ER stress contributes to the dynamic regulation of host–virus interactions and represents an important determinant of infection outcomes.

### 4.4. Metabolic Stress Signaling (AMPK–mTOR Axis) in Viral Infection

Cellular energy homeostasis is tightly regulated by the AMPK–mTOR signaling axis, which integrates nutrient availability with metabolic adaptation and biosynthetic control. During viral infection, this pathway plays a central role in coordinating host metabolic responses to the increased energetic and anabolic demands imposed by viral replication. Because glutamine is a key contributor to mitochondrial metabolism and biosynthetic processes, fluctuations in glutamine availability directly influence AMPK–mTOR signaling dynamics.

Glutamine deprivation impairs TCA cycle activity and reduces ATP production, leading to activation of AMPK, a major cellular energy sensor [10,29]. Activated AMPK promotes catabolic processes that restore energy balance while inhibiting anabolic pathways, primarily through suppression of mTOR complex 1 (mTORC1). In parallel, glutamine availability positively regulates mTORC1 activity through amino acid–dependent signaling mechanisms, including modulation of intracellular amino acid pools and lysosomal nutrient sensing pathways [29,30]. Thus, glutamine functions as both a metabolic substrate and a signaling regulator within the AMPK–mTOR axis.

During viral infection, modulation of AMPK–mTOR signaling exerts complex and context-dependent effects on viral replication. Activation of AMPK can restrict viral propagation by limiting biosynthetic capacity and inhibiting protein synthesis required for viral replication [72,73]. Conversely, some viruses exploit AMPK signaling to promote metabolic reprogramming and maintain cellular survival under stress conditions, thereby supporting sustained viral production [12,13,72]. Similarly, mTORC1 activity is frequently manipulated by viruses to enhance translation of viral proteins and support anabolic metabolism. For example, several viruses activate mTOR signaling to facilitate efficient viral protein synthesis and replication, whereas others transiently suppress mTOR to induce autophagy or adapt to nutrient stress conditions [14,30,74]. For example, the HCMV UL38 protein sustains mTORC1 activity under stress conditions by antagonizing the tuberous sclerosis complex (TSC), thereby promoting anabolic metabolism and efficient viral replication [75].

Glutamine metabolism further modulates these interactions by influencing both energy status and anabolic signaling. Enhanced glutaminolysis can support mTORC1 activation and biosynthetic pathways, thereby promoting viral replication. In contrast, glutamine insufficiency may shift the balance toward AMPK activation, leading to reduced anabolic activity and increased reliance on alternative metabolic pathways such as autophagy. This metabolic shift can either restrict viral replication or, in certain contexts, provide substrates that viruses can exploit.

The AMPK–mTOR axis is also closely integrated with other glutamine-linked stress pathways. AMPK activation promotes autophagy through phosphorylation of ULK1, while mTORC1 inhibition further facilitates autophagic flux. In addition, crosstalk with ISR and ER stress signaling coordinates translational control and metabolic adaptation under conditions of nutrient limitation. These interactions contribute to a highly dynamic regulatory network that is modulated during viral infection [72,74].

Importantly, the metabolic state of host cells prior to infection may influence AMPK–mTOR signaling responses and thereby affect viral susceptibility. Conditions of nutrient limitation, including glutamine insufficiency, can precondition cells toward an energy-conserving state characterized by AMPK activation and reduced mTOR activity. Such states may alter viral entry, replication efficiency, or host cell survival, depending on the specific virus and cellular context.

Collectively, the AMPK–mTOR signaling axis represents a critical interface between glutamine metabolism and viral infection. By integrating energy sensing with anabolic control, this pathway plays a key role in determining whether cellular conditions favor or restrict viral replication.

### 4.5. Autophagy in Viral Infection

Autophagy is a conserved catabolic process that maintains cellular homeostasis by degrading and recycling intracellular components under conditions of nutrient limitation and stress. Glutamine availability is a key regulator of autophagy through its effects on cellular energy status and redox balance. During viral infection, autophagy represents a critical interface between host adaptive responses and viral exploitation strategies [6,9,16,76].

Glutamine deprivation promotes autophagy primarily through activation of AMPK and inhibition of mTORC1, leading to ULK1 activation and initiation of autophagosome formation [77]. In addition, reduced glutamine-derived GSH enhances oxidative stress, which further stimulates autophagic signaling. These mechanisms position glutamine metabolism as an upstream regulator of autophagic flux under metabolic stress conditions.

In viral infection, autophagy exerts context-dependent effects. It can contribute to antiviral defense by degrading viral components and supporting immune responses. Conversely, many viruses exploit autophagic machinery to facilitate replication, for example by utilizing autophagic membranes for replication complexes or modulating autophagic flux to support viral assembly [16,76,78]. For instance, dengue virus NS4A protein has been reported to induce autophagy and remodel intracellular membranes, thereby promoting viral replication and cellular adaptation during infection [79,80]. Recent studies further emphasize the dynamic interplay between antiviral autophagy and virus-mediated exploitation of autophagic pathways during infection [81].

Glutamine-linked metabolic stress further influences these processes. Increased autophagy under glutamine insufficiency may provide alternative metabolic substrates that support cell survival and, in some cases, viral replication. Alternatively, excessive autophagy may contribute to antiviral restriction or cell death, depending on the cellular context and viral strategy.

Overall, autophagy functions as a downstream effector of glutamine-linked stress networks, integrating metabolic adaptation with host–virus interactions and contributing to the regulation of viral infection outcomes. Representative viruses exploiting glutamine-linked stress pathways during infection are summarized in Table 1.

## 5. Perspectives and Future Directions

Glutamine metabolism has emerged as a central regulator of cellular stress responses that influence viral infection. As discussed in this review, glutamine availability coordinates multiple interconnected pathways, including ISR, ER stress, AMPK–mTOR signaling, redox regulation, and autophagy [7,8,9,10,34,36]. Increasing evidence indicates that these glutamine-linked stress networks are actively engaged during viral infection and can exert both antiviral and proviral effects depending on the viral species, host cell type, and metabolic context.

Despite significant progress, several important questions remain unresolved. First, the precise mechanisms by which glutamine availability influences viral entry and early stages of infection are not fully understood. While most studies have focused on viral replication and protein synthesis, emerging observations suggest that metabolic stress conditions may also influence receptor expression, membrane organization, and virus–host interactions at the cell surface. Elucidating these early events will be critical for a comprehensive understanding of how metabolic states regulate viral susceptibility.

Second, the integration of glutamine metabolism with other nutrient-sensing pathways, particularly glucose metabolism, requires further investigation. Cellular metabolism operates as a highly interconnected network, and alterations in one nutrient pathway often affect others. The combined effects of glucose and glutamine availability on stress signaling and viral infection remain insufficiently characterized, especially in physiologically relevant cell types and tissue microenvironments.

Third, the role of physiological metabolic stress in specific developmental and tissue contexts warrants greater attention. Certain cell types, such as first-trimester trophoblasts, naturally experience low oxygen tension and limited nutrient availability. These conditions may predispose cells to a distinct metabolic and stress-response state that influences viral infection outcomes. Understanding how glutamine-linked stress pathways operate under such physiological conditions may provide important insights into tissue-specific susceptibility and disease pathogenesis.

From a translational perspective, targeting glutamine metabolism and associated stress pathways represents a promising strategy for host-directed antiviral therapy [12,55,73]. Pharmacological modulation of glutaminolysis, mTOR signaling, or autophagy has already been explored in other disease contexts, including cancer, and may be repurposed to modulate viral infection. However, given the dual roles of these pathways in both promoting and restricting viral replication, careful consideration of timing, dosage, and cell-type specificity will be essential to avoid unintended effects.

Future studies integrating metabolomics, single-cell analysis, and advanced imaging approaches will be instrumental in dissecting the dynamic interactions between glutamine metabolism and cellular stress responses during viral infection [9,12,34]. In addition, the use of physiologically relevant models, including primary cells and organoid systems, will help bridge the gap between in vitro observations and in vivo relevance. An additional challenge will be to understand the heterogeneity of glutamine-linked stress responses at the single-cell level during viral infection. Cellular metabolic states can vary substantially within infected tissues, potentially leading to distinct stress-response profiles and differential susceptibility to viral replication. Integration of single-cell transcriptomics, spatial metabolomics, and live-cell imaging approaches may therefore provide important insights into how metabolic heterogeneity shapes virus–host interactions and infection outcomes. In addition, further investigation of lipid ROS accumulation and ferroptosis-associated pathways may provide new insights into redox-dependent mechanisms linking glutamine metabolism, cellular stress responses, and viral infection [62,63,98].

In conclusion, glutamine-linked cellular stress responses represent a critical interface between host metabolism and viral infection. A deeper understanding of these processes will not only advance our knowledge of viral pathogenesis but also open new avenues for therapeutic intervention.

## Figures and Tables

**Table 1:** Representative viruses exploiting glutamine-linked stress pathways during infection.

Virus	Viral Type	Metabolic/Glutamine Reprogramming	Stress Pathways	Viral effector	Functional Outcome	References
HCMV	DNA	↑ Glutamine uptake; ↑ TCA cycle flux	mTOR; ER stress; autophagy	UL38	Supports biosynthesis and replication	[52,74]
Dengue virus	RNA	↑ Glutamine metabolism; ↑ lipid metabolism (lipophagy)	Autophagy; ER stress (PERK); ROS	NS4A/NS4B	Enhances replication complex formation	[51,82,83]
Influenza A virus	RNA	↑ metabolic reprogramming (primarily glucose; glutamine supportive)	AMPK; ROS	NS1	Supports replication	[54,73,84,85]
SARS-CoV-2	RNA	Altered metabolism; redox imbalance	ER stress; ISR; autophagy; GRP78	ORF8, NSP6	Modulates replication; enhances entry	[47,61,64,86]
Hepatitis B virus	DNA	Metabolic reprogramming; ↑ amino acid metabolism (including glutamine)	ER stress (UPR); ROS	HBx	Supports replication; promotes viral protein production	[87,88,89,90]
Hepatitis C virus	RNA	Reprograms amino acid (including glutamine, indirect) and lipid metabolism	ER stress, autophagy	NS4B, NS5A	Promotes replication and assembly	[9,91,92,93]
KSHV	DNA	↑ Glutamine metabolism (latency/reactivation)	mTOR; redox pathways	vGPCR/LANA (proposed)	Supports persistence and oncogenesis	[20]
ASFV	DNA	Redox remodeling; ↑ GSH	ER stress; PERK–eIF2α; ATF6–Ca^2+^; autophagy; ROS	EP152R, K205R	Favors replication environment	[66,67,68,69]
Rubella virus	RNA	Nutrient stress sensitivity; redox imbalance	ER stress; ROS; ISR (proposed)	Not clearly defined	Enhances infection; ↑ susceptibility	[15,19]
Rotavirus	RNA	↑ Glutamine catabolism; ↑ aspartate biosynthesis	ER stress; PERK–eIF2α; ROS; AMPK–Nrf2	NSP4	Supports replication; enhances biosynthesis	[94,95,96,97]

Abbreviations: HCMV, human cytomegalovirus; KSHV, Kaposi’s sarco-ma-associated herpesvirus; ASFV, African swine fever virus. ↑ indicates increased/upregulated activity or process.

## Data Availability

No new data were created or analyzed in this study.
